# Enjoying Poor Health

**Published:** 1873-10

**Authors:** Ben H. Pratt


### THE BISTOURY.

Vol. IX. ELMIRA, N. Y., OCTOBER, 1873. No. 3.

### Contributions.

### ENJOYING POOR HEALTH.

BEN H. PRATT, A. M.

There never was a time in the history of any age, so far as we can learn, when it apparently was so universally popular,—so pre-eminently proper—in fact so absolutely necessary to be out of health—as at present. One's physical condition is likely to become one of the most marked qualifications for, or objections to, a position in the so called Best Society of the time. That much ridiculed expression about "enjoying poor health," bids fair to express very patly the precise truth. This generation seems to "enjoy poor health" with a zest—no, that's too forcible—with a dreamy ecstasy that carries its sublimity home to the heart of the contemplative student of human nature. To be well is so vulgar. Not to have a debility, is so pronounced a defect as to excite genuine pity. It does seem so easy a thing to rake up an ailment, that he or she who fails to do so is either morbidly conscientious, or unconventionally bold, and is treated with the indifference that the heinousness of the fault deserves.

To be delicate is a positive evidence of the justice of one's claims to the highest social recognition. It gives such an air of *ennui*—superinduces such an interesting languor—affords such a never-failing topic of high toned conversation—is such a good excuse for dullness—so enables the most lack brained, witless and stupid soul to stand upon the same level with the more brilliant—is such a splendid leveler of genius and talent to the plane of nonentity and ignorance—so cunningly snatches the honors from the deserving and flings them into the ocean of sighs and complaints—that one knows not whether he is associating with animated compound hat racks and clothes pegs, or with speaking automatic lay figures of the dry goods shops.

This very large and very beaumondish class of folk, has brought its local organizations to a very complete issue. It holds its regular town or city meetings at its local Water Cure, from which it regularly sends its delegates to the County Spring of sonorous title, generally aboriginal, and thence, as commissioners, go forth its choicest spirits, chosen for their excess of etherealness, to the more pretentious Saratogan or Long Branch Congress, there to deliberate profoundly upon the programme of most fashionable and most irrational complaints to be adopted for the coming year. These deliberations of the debilitated are the acme of all hygienic wisdom, and exhibit a profound knowledge of the subject that shows how closely its applications to the cases of those interested has been studied.

It is somewhat difficult for those in merely comfortable circumstances to fully appreciate the luxury of delicate health. We who are accustomed to congratulate ourselves when we feel very well indeed, and consider ourselves peculiarly unfortunate when our functions do not all act in a normal fashion, cannot conceive of any one's enjoying that which is painful or in the slightest degree irregular in the matter of health. That such a condition should be sought after, and if not really attainable, simulated, is still more surprising. But when we remember that there is not a folly on earth that is not advocated by more or less of every community, and as a general rule, the greater the folly the more followers it obtains, it ought not to excite our wonder.

So far as the object sought after by the great company of those who annually visit our fashionable watering places is concerned, a great error universally prevails. It is to correct that misapprehension that we entered upon this article.

Having stated above that health is not desirable by a very large proportion of our people; and that under the ordinary circumstances of life mankind is apt to attain to, and remain in-a state of health that is incompatible with the views of a sect of society, the ultra fashionable; it becomes a vexed question with those healthful ones how to attain to a sufficient status of delicacy to warrant their admission to, and continuance in that fashionable organ, ization, which we can designate by no better term than the etherealists. The mode of life adopted at our summer resorts is especially framed with this object in view, and we cannot sufficiently admire the ingenuity of the designers thereof. They have ransacked every department of hygienic science, and carefully culled therefrom all the elements that are deleterious to the maintenance of good health, at the same time carefully avoiding all the precepts of the best thinkers and writers upon the best modes of procuring lost, and sustaining regained health. A cursory examination of the routine of a fashionable summer resort will bear us out in our assertions.

The first sensations one experiences at a Summer Hotel are those of absolute freedom from all restraint. If a man, he has no cares —no business—no duties. Accustomed to having these, he naturally misses them—looks for them. They have been part and parcel of his life, and he has learned to depend upon them for so much of excitement as nature deems it necessary that every man should have. The lack of these actually worries, and the man of business really begins to tire of his recreation ere it has begun, and to wish him. self back among the actualities of his business pursuits. Thus a void exists in his nature, demanding to be filled, and he fills it with that which is presented him first, in the way of substitute for something better. He drinks, if so inclined, to excess—he eats, for the lack of something else to do, immoderately, and of the most highly seasoned and dainty food, in lieu of a lack of natural appetite, and having so eaten, resorts to artificial means of producing digestion. Gaming is a temptingly displayed relief for discomforts of all sorts, and one most frequently resorted to, and the ease with which it can be carried to an injudicious extent, is a fact to which any *habitue* of these places will bear witness. Undue excitement and a derangement of the stomach, effect morbid conditions of body very quickly, and our friend soon begins to assume an air that betokens his growing eligibility to a prominent place among the delicate ones. If a woman, her household and family duties have been set aside, and eating and dressing and small talk, with little or no exercise, have their accustomed effect upon her temperament and health. With both the sexes, exceedingly late hours become the rule, and dissipations that at home would be regarded as not only injudicious, but even outrageous, are indulged without compunction. Nights spent between the ball room and the dining room, and the forenoons given up to fitful slumber —the complete unnaturalness of every feature of the life so led, and the entire abnegation of long formed habits—if these fail to undermine the stoutest constitution, they at least afford a rich soil for the growth of the seeds of disease thus planted. The children, however, are the worst sufferers, for though they are the willing, they are not the culpable participators in the life they lead. The examples of men and women among whom they are cast, and to whom they naturally look for something better and wiser than one often finds at these resorts, have an influence that is not for good, either in a physical or moral sense. Improper liaisons, too, are the natural outgrowth of such a promiscuous community as must necessarily assemble under a single roof, where a lack of regular duty affords time, and necessarily intimate associations bring about opportunities that are temptingly immoral in their tendencies.

The whole system of summer living in summer hotels at summer resorts, is baneful to the physical health and to the moral sense, besides inculcating a false idea of the stern business of real life, clothing its men and women in a garment of sham, and snatching from one's nature the idea which is as true and eternal as creation, that work of some sort is due from every human being that has been given an existence upon the earth. Besides the bad effects, both to health and morals, we contend that the sort of habits engendered by a sojourn at a watering place is injurious to the best interests of the community. It gives birth to, and fosters a notion of an ideal existence that unfits one for the actuality that surrounds him in his daily dealings with the world.

It is also an injurious extravagance for the many of moderate means, who strive to emulate the wealthy few in all the expensive accessories of life at a watering place. The result of one or two reasons of this fashionable folly has been bankruptcy in several instances we know of. The fact has been admitted to us privately by more than one victim, that the beginning of financial embarrassment was a useless trip to a noted resort—a trip taken only because the family had not the moral courage to defy the requirements of their set, even in the face of the fact that such an extravagance must necessarily jeopardize their prospects in the future.

The financial, social, and moral effects of this folly it was not our purpose to touch, at the outset of this article. We have alluded to them as simply attendant evils upon the growing custom. The real harm done grows out of a mistaken idea that health is benefitted by a sojourn during the warmer months at a place where all the laws of health are ignored. The custom is being carried to an injurious extent, and perhaps the only way to effect a reform is to allow the mania to run its course unchecked until it reaches a *reductio ad absurdum*—a point where its flimsiness will become so glaringly apparent as to challenge the attention, and meet the reprobation of the shallowest minds.

But there is a recreation that is worthy of adoption. It is not fashionable—not expensive—not exciting—not desirable for those in pursuit of poor health. It is vulgarly healthful, however, and a promoter of those primitive ideas of life and manners which are ignored by too many of the present day.

If, during the summer, or a portion of it, one have leisure, let him engage a home in the family of some farmer whose broad fields and sterling good sense are known. Let him close up his house and gathering together his household, take them, with only so much of clothing as cleanliness and decency demand, to the country. Let him ransack the farm in company with the men; let his wife explore the mysteries of the kitchen and dairy, not as a visitor but as a familiar friend; let the children roam the fields and romp the country over. It may be thought tame sport by the city bred, when the proposition first reaches him, but a trial will satisfy the most incredulous that there is no higher type of enjoyment—no greater change from accustomed habits—no more complete recreation than a good sensible summering in the humble though hospitable home of a well-to-do, thrifty, busy farmer.

